# Acute mesenteric ischaemia: a pictorial review

**DOI:** 10.1007/s13244-018-0641-2

**Published:** 2018-08-17

**Authors:** S. Florim, A. Almeida, D. Rocha, P. Portugal

**Affiliations:** Department of Radiology, Centro Hospitalar Vila Nova de Gaia/Espinho, Rua Conceição Fernandes, 4434-502 Vila Nova de Gaia, Portugal

**Keywords:** Mesenteric ischaemia, Computed tomography, Pneumatosis intestinal, Ischaemia/diagnosis, Mesenteric vascular occlusion/diagnosis

## Abstract

**Abstract:**

Acute mesenteric ischaemia (AMI) is an uncommon cause of acute hospital admission with high mortality rates (50–90%) that requires early diagnosis and treatment. With the increase in average life expectancy, AMI represents one of the most threatening abdominal conditions in elderly patients. Untreated, AMI will cause mesenteric infarction, intestinal necrosis, an overwhelming inflammatory response and death. Early intervention can reverse this process leading to a full recovery, but the diagnosis of AMI is difficult. The failure to recognise AMI before intestinal necrosis has developed is responsible for the high mortality of the disease. Unfortunately, common CT findings in bowel ischaemia are not specific. Therefore, it is often a combination of nonspecific clinical, laboratory and radiological findings that helps most in the correct interpretation of CT findings. The purpose of this article is to provide an overview of the anatomy, physiology of mesenteric perfusion and discussions of causes, pathogenesis and CT findings in various types of acute bowel ischaemia. Familiarity with various imaging features of mesenteric injury is essential to make a timely diagnosis that will lead to improved patient outcomes.

**Teaching Points:**

• *AMI is a potentially life-threatening disorder whose prognosis depends on early recognition, accurate diagnosis and timely intervention*.

• *Arterial inflow occlusion due to thrombosis or embolisation is the most common cause of AMI*.

• *Four aetiological types of AMI have been associated with different characteristics and risk factors (EAMI, TAMI, VAMI and NOMI)*.

• *Physical examination and laboratory findings are not sensitive or specific for diagnosing AMI; therefore, MDCT is still the first-line imaging method in suspected AMI*.

• *Although a number of scoring systems for prognosis have been proposed, these have not been validated in large-scale studies*.

## Background

Intestinal ischaemia refers to insufficient blood flow within the mesenteric circulation to meet the metabolic demands in the bowel. It is a potentially catastrophic entity that may require emergent intervention in the acute setting. Although the clinical signs and symptoms of intestinal ischaemia are nonspecific, CT findings can be highly suggestive in the correct clinical setting.

## Anatomy and pathophysiology

**Fig. 1:**
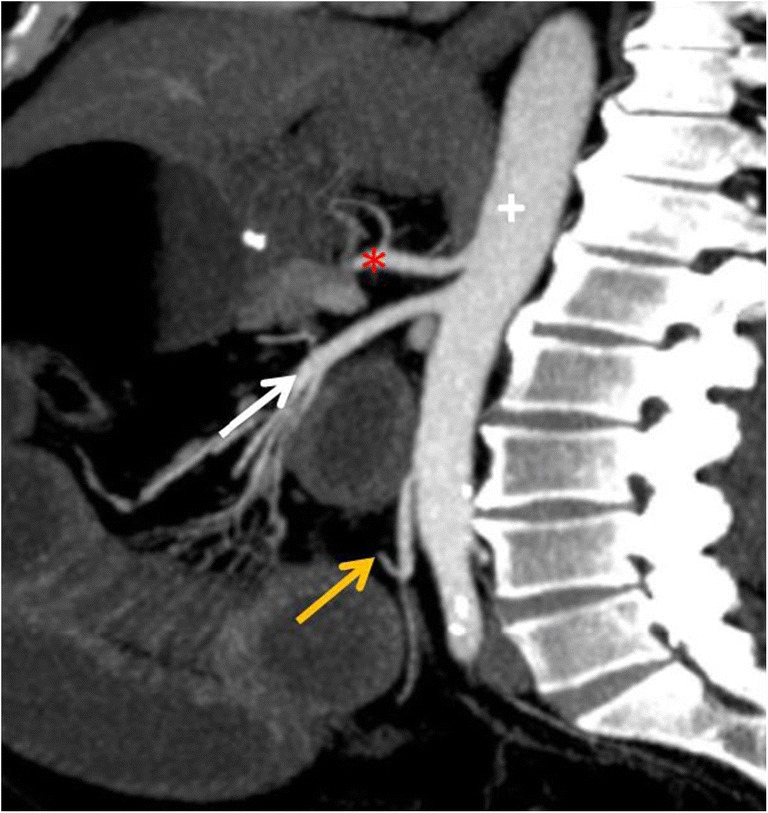
Anatomy. Sagittal CT MIP (maximum intensity projection) image shows three major arteries that supply the bowel, coeliac trunk (asterisk), superior mesenteric artery (white arrow) and inferior mesenteric artery (orange arrow), which are visceral branches of the abdominal aorta (+)

To accurately diagnose mesenteric ischaemic disease, the radiologist must be acquainted with both the mesenteric arterial and venous anatomies of the bowel. The three major arteries that supply the small and large bowel are the coeliac trunk, superior mesenteric artery (SMA) and inferior mesenteric artery (IMA) (Fig. 1). The coeliac trunk generally provides the blood supply to the distal oesophagus to the second portion of the duodenum. The superior mesenteric artery supplies the third and fourth portions of the duodenum, jejunum, ileum and colon to the level of the splenic flexure. The IMA supplies the distal colon from the level of the distal transverse portion to the upper rectum.

Middle and inferior rectal arteries, branches of the internal iliac arteries, supply the distal rectum.

There are numerous important mesenteric collateral pathways that provide a rich vascular safety net for the mesenteric blood supply. The gastroduodenal artery, usually the first branch of the common hepatic artery, provides an important collateral pathway between the coeliac artery and SMA. The marginal artery of Drummond (marginal artery of the colon) and arcade of Riolan (intestinal arterial arcade) connect the SMA and IMA. Four arcades of anastomosis are formed between the IMA and lumbar arteries arising from the aorta, sacral and internal iliac arteries. In addition, the peripheral small mesenteric vessels are anatomically arranged in a parallel series configuration that supplies the mucosa, submucosa and muscularis propria of the bowel [1].

The venous system returns essentially parallel to the arterial supply. The superior and inferior mesenteric veins run parallel to the arteries and drain the respective part of the bowel. The inferior mesenteric vein (IMV) joins the splenic vein, and the splenic vein joins the superior mesenteric vein (SMV) to form the main portal vein. Numerous collateral venous pathways can form between the mesenteric and systemic veins including the gastric and oesophageal, renal, lumbar and pelvic veins.

Under normal circumstances, the human bowel receives around 20% of the resting cardiac output, of which two-thirds supplies the intestinal mucosa [2, 3]. In the postprandial phase, splanchnic autoregulation may increase intestinal blood flow to as much as 35% of cardiac output.

Autoregulation of intestinal perfusion may maintain tissue viability below a systemic blood pressure of 70 mmHg; however, in cases where systemic blood pressure is below 40 mmHg, local myogenic autoregulation is overruled by systemic autoregulation and local protective mechanisms fail. This results in an increasingly ischaemic bowel wall, similar to what would happen in the case of mesenteric underperfusion due to vascular occlusion [4, 5].

The initial ischaemic damage to the intestinal wall may then range from only mild and superficial necrosis limited to the mucosa (stage I), or damage extending to the submucosal and *muscularis propria* layers (stage II), to dangerous and life-threatening continuous necrosis of all bowel wall layers (transmural bowel infarction) (stage III) [6, 7]. The first two stages are poorly identified on CT.

From the pathophysiological point of view, the initial purely ischaemic lesions of the intestine are typically followed by the release of certain mediators such as cytokines, platelet-activating factor and tumour necrosis factor, which will lead to an inflammatory response and additionally damage the bowel wall [8, 9]. As a consequence of several hours of transmural necrosis, the mucosal barrier breaks down and the bowel loses its resistance to bacterial invasion, leading to bacteraemia and sepsis [10].

## Aetiology and medical history

Mesenteric ischaemia can be of acute (90%) or chronic type (10%). In acute type the causes are arterial embolism, arterial thrombosis, nonocclusive form or venous occlusion.

Acute occlusions of the superior mesenteric artery due to thrombosis or embolisation are responsible for approximately 60%–70% of cases of acute bowel ischaemia, whereas nonocclusive conditions account for approximately 20%–30% of cases and mesenteric venous thromboses account for 5%–10% of the total [10, 11].

### Embolic and thrombotic acute mesenteric ischaemia (EAMI/TAMI)

**Fig. 2:**
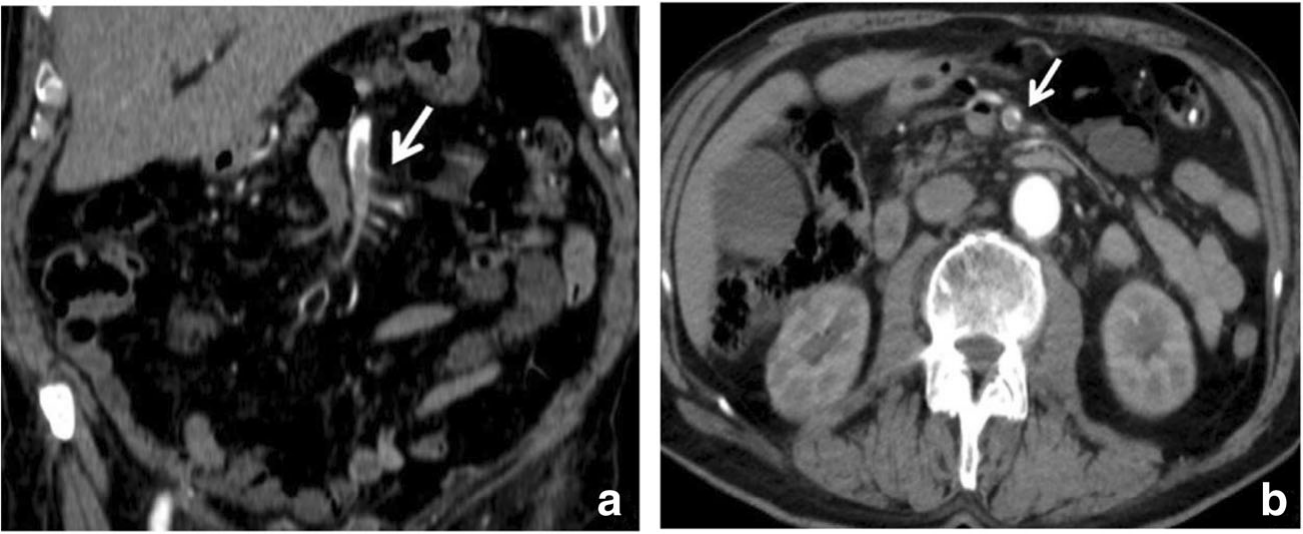
Arterial occlusion. Contrast-enhanced CT image of the abdomen in a 54-year-old female with superior mesenteric arterial thrombosis. **a**, **b**: Contrast-enhanced axial CT scan demonstrates a thrombus in the superior mesenteric artery in coronal (**a**) and axial plane (**b**) (white arrow)

Arterial inflow occlusion most commonly results from thromboembolism, where the embolus originates from the left atrium as a consequence of atrial fibrillation (Table 1). Emboli preferentially affect SMA because of its small take-off angle compared with those of the coeliac and IMA. While thrombi and large emboli may occlude the proximal SMA and ostia of major mesenteric vessels resulting in extensive small bowel and colon ischaemia, smaller emboli may lodge in the distal portions of the vessel and cause smaller regions of segmental ischaemia [12, 13]. Acute arterial thrombi and emboli may appear as obvious low-attenuation filling defects in the luminal vessels (Fig. 2).

**Table 1:** Causes of IMA by aetiology

Arterial occlusion	Venous occlusion	Nonocclusion
Arrhythmia	Infiltrative	Hypovolaemia
Myocardial infarction	Neoplastic	Hypotension
Valve disease	Inflammatory (may encase mesenteric veins)	Low cardiac output
Atherosclerosis	• Acute pancreatitis	Digoxin
Prolonged hypotension	• Appendicitis	α-Adrenergic agonists
Spontaneous isolated superior mesenteric artery	• Diverticulitis	Dysautonomy syndrome
Dissection (SISMAD)	• Peritonitis	Pheochromocytoma
Occlusions of large vessels:	Hypercoagulability state:	High-endurance athletes
• Takayasu arteritis	• Polycythaemia vera	Trauma
• Giant cell arteritis	• Sickle cell disease	Radiation
Occlusions of medium vessels	• Thrombocytosis	Corrosive injury
• Panarteritis nodosa	• Antithrombin III	Immunosuppression
• Kawasaki disease	• Protein C/protein S deficiencies	Chemotherapy
Occlusions of small vessels:	• Carcinomatosis	Pharmaceutical agents that lead to mesenteric vasoconstriction
• Systemic lupus erythaematosus	• Pregnancy	• Digitalis
• Schonlein-Henoch purpura	• Oral contraceptives	• Vasopressin
• Wegener granulomatosis	Right-side heart failure	• Adrenaline
• Churg-Strauss syndrome	Enterocolic lymphocytic phlebitis	• Noradrenaline
• Buerger disease		• Ergotamine
• Rheumatoid vasculitis		Pharmaceutical agents that lead to hypotension:
• Behcet syndrome		• Antihypertensive drugs
Thrombotic thrombocytopaenic purpura		• Diuretics
Haemolytic uraemic syndrome		• Neuroleptics
Fibromuscular dysplasia (children++)		• Antidepressants
		Inhibition of prostaglandin synthesis:
		• Indomethacin
		Drugs:
		• Cocaine
		• Crack cocaine
		• Heroin

There are various causes of occlusion of the mesenteric arteries besides embolism (Table 1). In younger patients thrombotic microangiopathies and antiphospholipid antibody syndrome are also frequent causes of occlusion of the mesenteric arteries.

The development of intestinal ischaemia from an arterially obstructing lesion depends upon the location of the obstruction, the patient’s collateral vasculature, acuity and degree of the obstruction. As said before, the presence of collateral arcades allows bidirectional flow, which can bypass obstructing lesions. In the presence of obstructions involving all three major arteries (coeliac, SMA and IMA), the phrenic, lumbar and pelvic collateral arteries may dilate to provide accessory visceral blood flow. However, if the lesion is distal to the point of collateral flow, the collateral supply is ineffective and ischaemia is more likely to ensue.

Clinically, EAMI is characterised by a sudden onset of abdominal pain in patients over 70 years old and a history of atrial fibrillation. In the early course of the disease, it can be characterised by an initial discrepancy between the severity of abdominal pain and minimal findings on physical examination. Patients can also present with symptoms of nausea, vomiting and initial forced evacuation. The location of pain varies, but as ischaemia progresses to infarction, it becomes diffuse and signs of peritoneal irritation appear. The development of transmural infarction may also be signalled by fever, bloody diarrhoea and shock.

Thrombotic arterial mesenteric ischaemia (TAMI) has a more indolent course. TAMI patients undergo gradual progression of arterial occlusion; therefore, many report symptoms of mesenteric angina (postprandial abdominal pain lasting up to 3 hours and nausea), which results in “food fear”, early satiety and weight loss. In the acute setting, however, the clinical symptoms are similar to those found in patients with EAMI [14].

The main risk factors for TAMI are atherosclerotic disease and dyslipidaemia [15]. There may be a history of other vascular events and previous vascular surgery.

### Venous acute mesenteric ischaemia (VAMI)

Mesenteric venous thrombosis (Fig. 4) may be caused by infiltrative, neoplastic or inflammatory/infectious conditions [7].

VAMI appears in younger patients, over 40, sometimes with several days of mild symptoms. Although occasionally idiopathic, up to 50% of patients have an identifiable risk factor, such as previous deep venous thrombosis or pulmonary embolism [16–18]. Other risk factors include a hypercoagulability state such as Leiden factor V mutation, oral contraceptive use, cirrhosis and advanced malignancy [19].

As in arterial occlusion, isolated proximal mesenteric venous thromboses usually do not lead to severe bowel ischaemia because of the extended collateral network between the mesenteric and systemic veins. In contrast, thrombosis of very distal mesenteric veins usually leads to severe haemorrhagic infarction of the bowel wall.

The onset of VAMI is characterised by subacute abdominal pain that may develop over a period of up to 2 weeks. VAMI is not usually associated with postprandial syndrome, although bloating, abdominal distension, fever and occult blood in stools may be present [20].

### Non-occlusive mesenteric ischaemia (NOMI)

In the setting of non-occlusive causes such as septic, haemorrhagic or cardiogenic shock, a profound drop of systemic blood pressure results in a reflexive mesenteric arterial vasoconstriction with diversion of blood flow to the brain and heart. As a consequence, intestinal perfusion will decrease dramatically and nonocclusive bowel ischaemia may develop.

Risk factors for NOMI include age over 50, history of acute myocardial infarction, congestive heart failure, aortic insufficiency, cardiopulmonary bypass, kidney or liver disease or major abdominal surgery. Notably, many patients with NOMI may have none of these factors [2].

The diagnosis of NOMI is the most challenging, first because it is often silent as it occurs in patients that are critically ill and often ventilated and second because CT findings overlap with those of other forms of bowel disease such as infectious and inflammatory enteritis and colitis [21–23].

The diagnosis should be suspected in patients with mesenteric hypoperfusion secondary to circulatory shock or vasoactive drugs when there is a significant unexpected deterioration in their clinical course [21, 24]. Acute or insidious pain (without defecation), bloating, abdominal distension and the presence of occult blood in the stools are all consistent with NOMI in a critically ill patient.

There has been an overall decrease in the incidence of this syndrome with improved management of haemodynamic instability.

## Diagnosis

Physicians are faced with a broad differential diagnosis in patients with abdominal pain. As physical examination findings and the most common laboratory abnormalities found in AMI are not sufficiently sensitive or specific to diagnose it [25, 26], researchers are still looking for the ideal plasma biomarker [27].

Lactic acidosis, which was considered an important laboratory finding because it indicates anaerobic metabolism, today is not a useful tool because of its lack of specificity. It develops late in the course of AMI when there is an extensive transmural infarction and at that point mortality is already 75% [28]. Therefore, MDCT has a high specificity and sensitivity [29] and should be the first-line imaging method in suspected AMI because of its high diagnostic accuracy [30, 31] and ability to exclude other causes of acute abdominal pain [8].

In this context, triphasic CT involves the acquisition of scans in the pre-contrast, arterial and venous phases. Unenhanced CT should always be performed prior to contrast-enhanced CT to detect vascular calcification, hyper-attenuating intravascular thrombus and intramural haemorrhage. Pre-contrast scans are also important so that differentiation among acute intramural haemorrhage, hyperaemia and hyperperfusion can be made.

Contrast-enhanced CT allows the identification of thrombus in the mesenteric arteries and veins, abnormal enhancement of the bowel wall and the presence of embolism or infarction of other organs. MDCT images should be obtained from the dome of the liver to the level of the perineum to cover the entire course of the intestine. Sagittal reconstructions are used to assess the origin of the mesenteric arteries [32].

The use of oral contrast is not recommended in patients with AMI. The transit time for oral contrast through the bowel will delay definitive treatment in AMI and the associated vomiting and adynamic ileus limit the useful passage of oral contrast material [32, 33].

Percutaneous angiography has been replaced as the gold standard for diagnosis of suspected AMI by MDCT. No relevant trials supporting the use of angiography for diagnosis can be found in the recent literature [34]. Currently, it is a second-line imaging modality that can be extended to relieve areas of obstruction in the mesenteric arteries [35].

## Ischaemic colitis: an important differential diagnosis

**Fig. 3:**
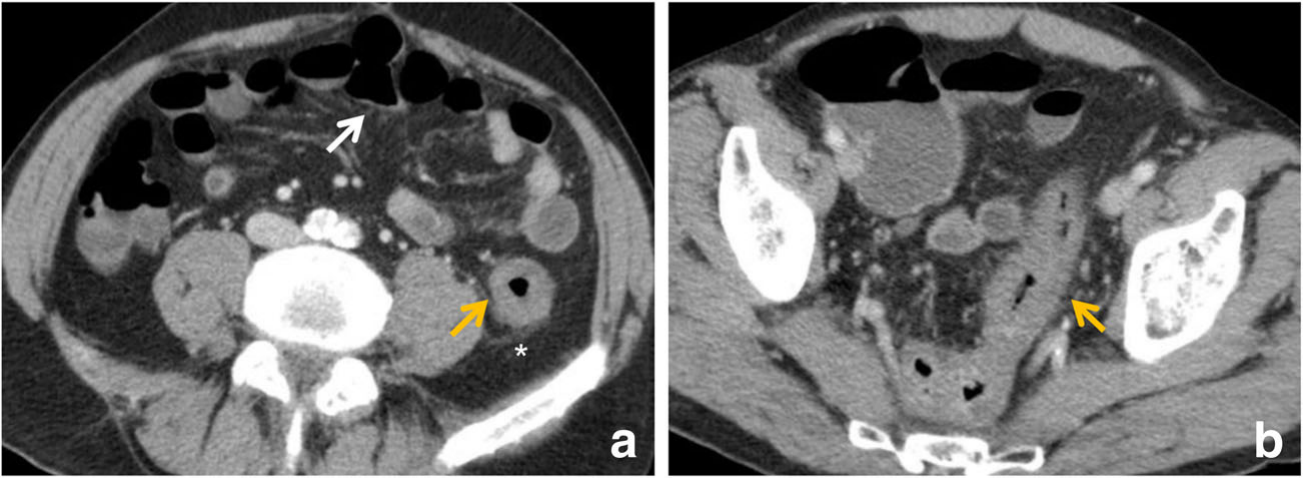
Ischaemic colitis in a 74-year-old male with vasculitis who presented with abdominal pain and bloody diarrhoea. (**a**, **b**) Contrast-enhanced CT scan reveals involvement of the sigmoid colon and splenic flexure (orange arrows) with marked wall thickening and pericolic streakiness (asterisk). Diagnosis was confirmed at colonoscopy and biopsy. The ischaemic process resolved

Although it was not one of the original goals of this article to review ischaemic colitis, we regard distinguishing the two entities that are often interchanged important (Fig. 3).

Even though ischaemic colitis and mesenteric ischaemia share several aetiological factors, colitis often has a more insidious onset; symptom onset usually hours while mesenteric ischaemia is sudden, the loss of arterial blood supply is only transient, clinically the pain is moderate and it also includes an associated sanguineous diarrhoea. Its treatment is usually conservative contrary to that of mesenteric ischaemia [36].

## Key CT key diagnostic findings: What to look for

Acute mesenteric ischaemia reveals various morphological and attenuation abnormalities on CT images in the bowel wall, mesenteric vessels and mesentery.

These different presentations depend on the pathogenesis of bowel ischaemia, site, extent of the ischaemic attack as well as the state of the collateral circulation, acuteness, duration, presence of superimposed bowel wall infection or bowel wall perforation.

Imaging findings in acute bowel ischaemia can be divided into imaging findings related to the bowel wall and extra-bowel wall signs.

## Bowel wall evaluation

### Bowel wall thickness

Normal bowel wall thickness ranges from 3 to 5 mm, although this strongly depends on the degree of bowel distention [37, 38].

Although it is not a specific finding, bowel wall thickness is the most common CT finding in acute bowel ischaemia. It is present in 26%–96% of reported cases [39].

**Fig. 4:**
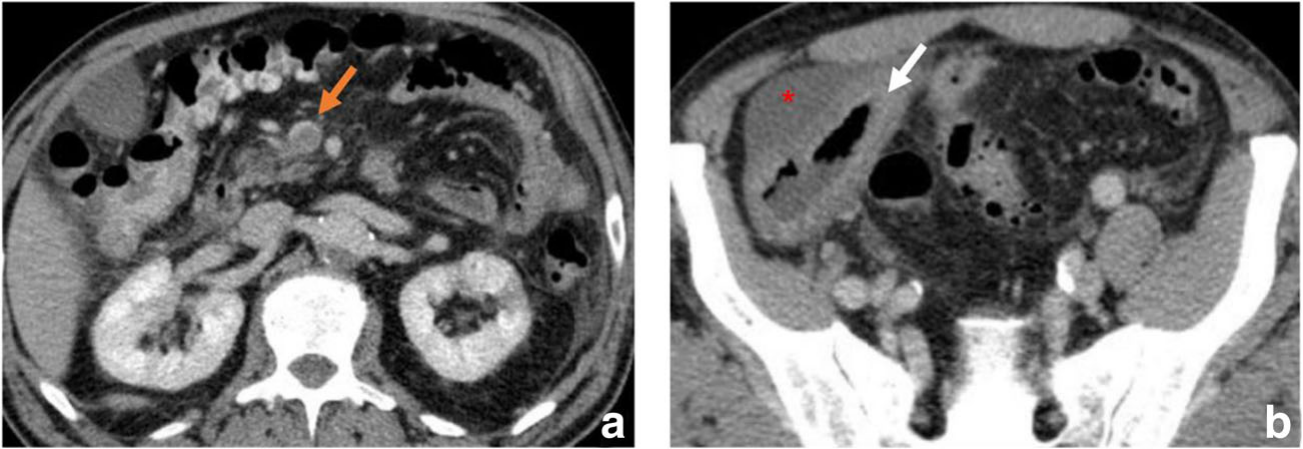
Venous occlusion. Contrast-enhanced CT image of the abdomen in 49-year-old female with superior mesenteric vein thrombosis. (**a**) Contrast-enhanced axial CT scan shows a thrombus in the superior mesenteric vein in axial plane (orange arrow); (**b**) wall thickening of the ascending colon (white arrow). Ascites (*) is also visible

In AMI, the bowel wall may be thickened or thinned, depending on the aetiological mechanism. In cases of bowel ischaemia caused by occlusions of mesenteric veins, bowel wall thickening is more pronounced than in cases caused exclusively by occlusions of mesenteric arteries (Fig. 4). This is caused by haemorrhage, oedema and/or superimposed infection, which results in bowel wall thickening of up to 15 mm of the small and large intestines [31].

**Fig. 5:**
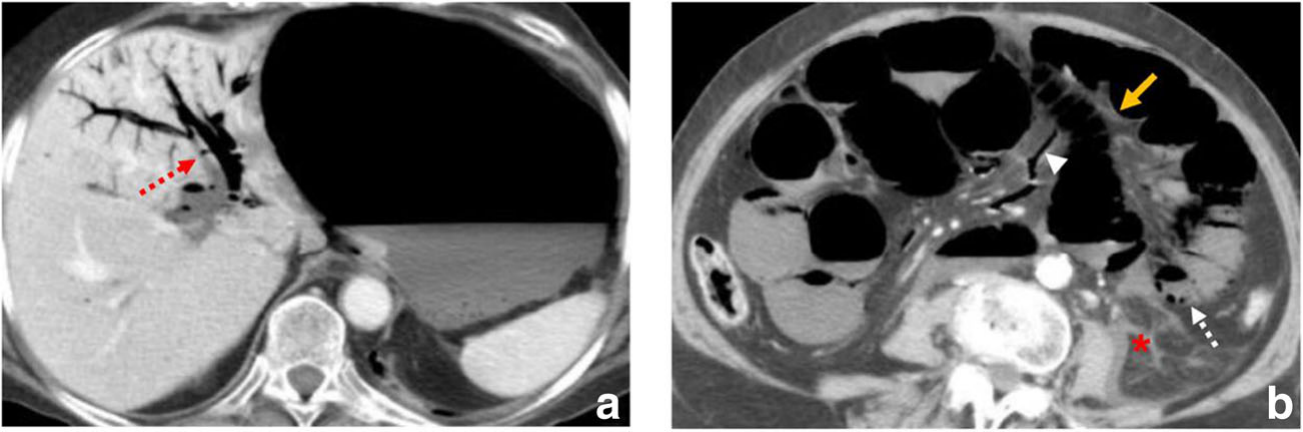
Contrast-enhanced CT image of the abdomen in an 82-year-old male with embolism of the superior mesenteric artery. (**a**) Contrast-enhanced axial CT shows pronounced intrahepatic portal venous gas (branching hypoattenuating areas) extending into the periphery of the left liver lobe (red arrow). (**b**) Contrast-enhanced axial CT scan shows dilated and gas-filled loops with an extreme thinning of the bowel wall, a “paper thin wall”, due to transmural small-bowel infarction (orange arrow). Pneumatosis (white arrow) is also seen; fat stranding (*) and gas in mesenteric veins (white arrowhead)

In cases of arterial occlusion or transmural infarction, the intramural nerves and intestinal musculature may be destroyed; therefore, infarcted bowel segments typically show dilated and fluid-filled or gas- and fluid-filled loops with an extreme thinning of the bowel wall, a “paper thin wall” (Fig. 5), due to total loss of tone and dilatation. Otherwise, in cases of reversible ischaemia, only mild bowel thickening may be noted [37].

In summary, the presence of bowel wall thickening is a common radiological sign of AMI, but the degree of thickening does not correlate with the severity of ischaemic bowel wall damage.

### Bowel dilatation

Luminal dilatation and air-fluid levels are quite common in acute bowel infarction (56%–91% of cases).

Bowel dilatation may result from interruption of intestinal peristalsis as a reflex to ischaemic injury or from irreversible and transmural ischaemic damage to the bowel wall. In the colon when only mild mucosal ischaemia occurs, a still viable bowel segment leads to spastic contractions; therefore, bowel dilatation is not usually seen.

**Fig. 6:**
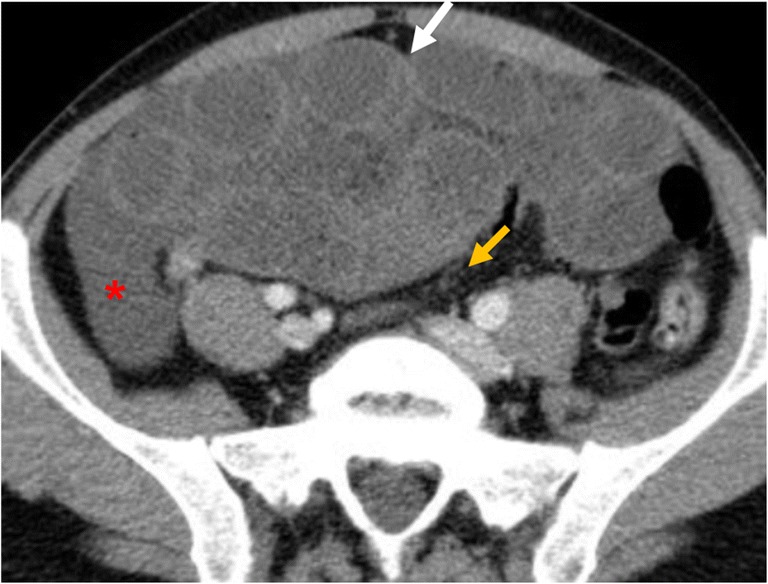
Gasless abdomen. Contrast-enhanced CT image of the abdomen in a 62-year-old female with small bowel wall ischaemia due to venoocclusive disease. Contrast-enhanced CT image of the abdomen shows dilated and mainly fluid-filled bowel loops and gasless abdomen (white arrow). Ascites (*) and vascular engorgement (orange arrow) are also seen

In small bowel wall ischaemia caused by venoocclusive disease, the presence of dilated and mainly fluid-filled bowel loops results from additional fluid exudation into the lumen of ischaemic bowel segments (gasless abdomen) (Fig. 6). In exclusive arterial occlusion, the dilated bowel loops seldom contain fluid-filled loops.

### Bowel wall attenuation

An ischaemic bowel segment may manifest with a hypoattenuating or spontaneous hyperattenuating bowel wall.

This is why we should always make assessments on both unenhanced and contrast-enhanced CT images to avoid misinterpretation of high density of the bowel as normal enhancement on contrast-enhanced CT in cases of intramural haemorrhage.

On unenhanced CT images, hypoattenuation of the bowel wall indicates bowel wall oedema, which is more typical in cases of acute bowel ischaemia caused by mesenteric venous occlusions. Otherwise, under these circumstances, hyperattenuation of the thickened bowel wall could also be seen because of intramural haemorrhage and haemorrhagic infarction.

**Fig. 7:**
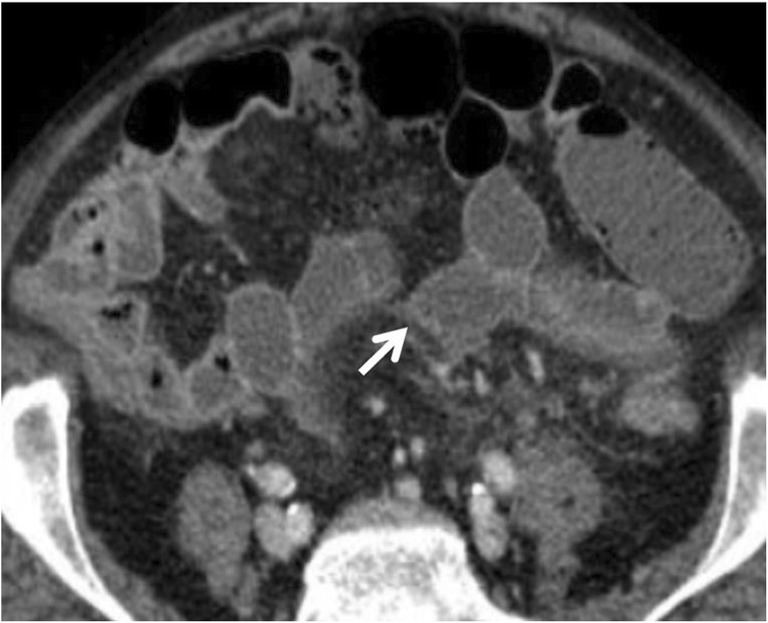
Bowel wall enhancement. Contrast-enhanced axial CT scan shows normal dilated loops with a diffuse hypointense segment of small bowel wall (white arrow) with reduction of bowel wall thickness due to superior mesenteric artery occlusion

**Fig. 8:**
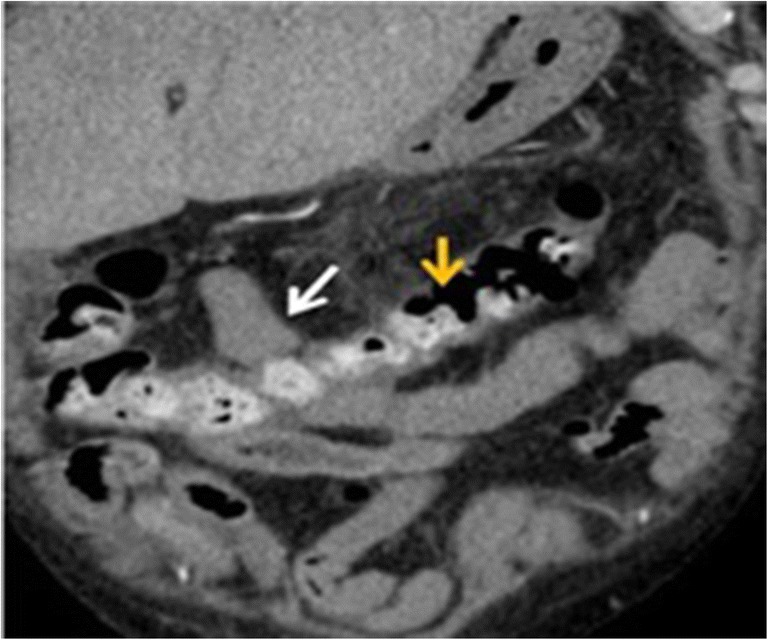
Bowel wall enhancement. Coronal contrast-enhanced CT shows “pale arterial ischaemia” with absent mural enhancement in a segment of small bowel (white arrow). An adjacent segment of small bowel shows mucosal hyperenhancement of thick-walled small bowel (orange arrow), indicating bowel reperfusion injury

**Fig. 9:**
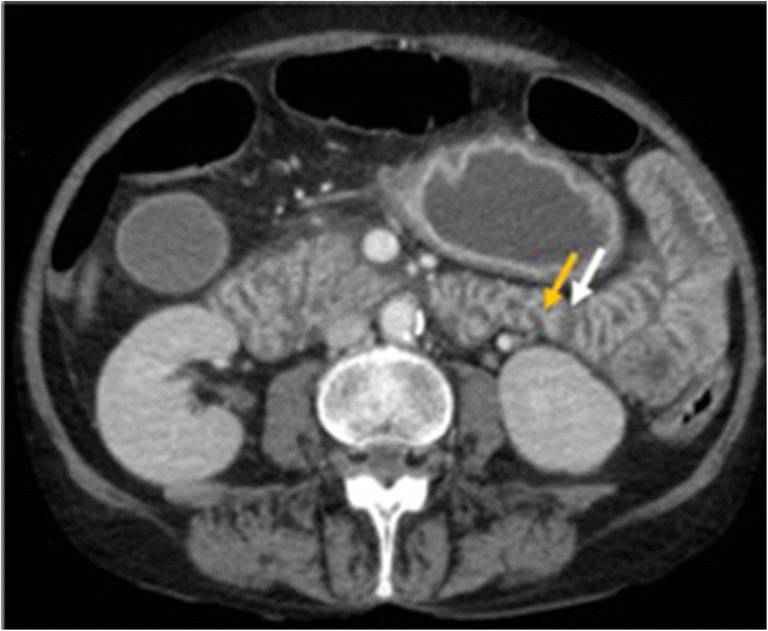
Shock bowel. Contrast-enhanced axial CT images in 56-year-old male with severe head trauma and bleeding from a large scalp laceration. Findings of shock bowel include diffuse intense mucosal enhancement (orange arrow) and submucosal oedema (white arrow)

On contrast-enhanced CT images, a highly specific although not sensitive finding for AMI is absent or substantially diminished bowel mural enhancement and has been termed “pale ischaemia” (Figs. 7 and 8). Alternatively, in the postreperfusion period after arterial injury, hyperenhancement of the bowel is present [40] much like “shock bowel” (Fig. 9).

Two factors that may cause hyperattenuation of ischaemic bowel walls on contrast-enhanced CT scans are hyperaemia and hyperperfusion of the bowel wall.

**Fig. 10:**
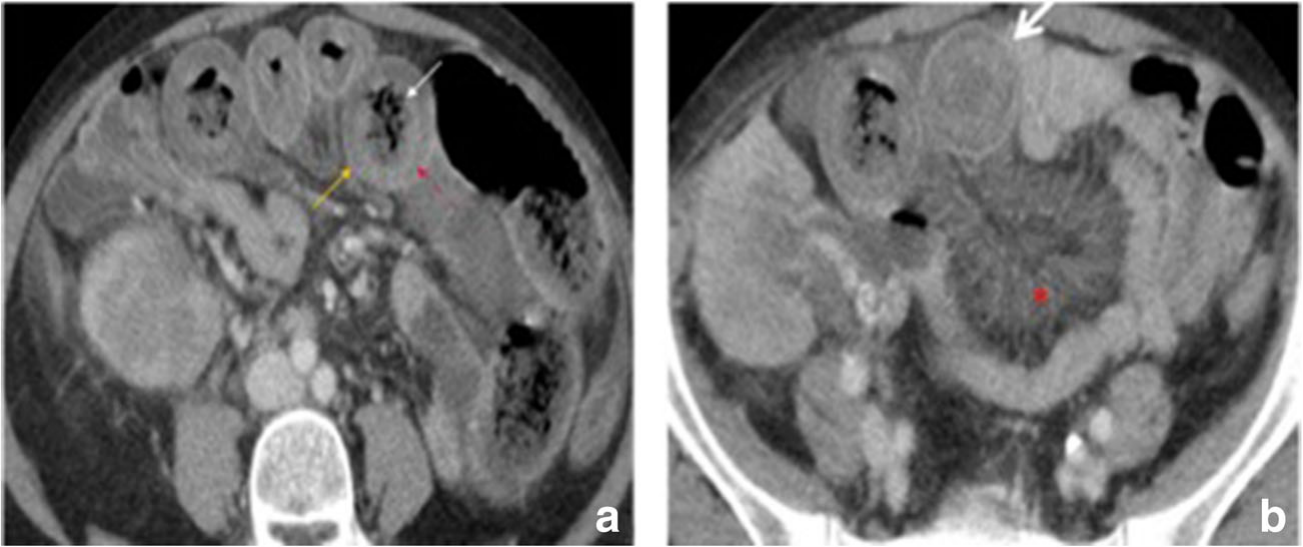
Bowel wall thickness and enhancement. **a** Contrast-enhanced axial CT images show a target sign (larger white arrow) in the small bowel wall due to mesenteric venous occlusion. **b** The vascular engorgement (*) and oedema of the bowel wall in turn lead to leakage of extravascular fluid into the bowel wall and mesentery. The resultant oedematous bowel may have a “halo” or “target” appearance due to mild mucosal enhancement (straight white arrow), submucosal and muscularis propria nonenhancement (red arrow), and mild serosal/subserosal enhancement (orange arrow). Bowel wall thickness is greatly increased measuring up to 1.5 cm

Hyperaemia of ischaemic bowel segments without hyperperfusion typically occurs in cases of mesenteric venous occlusion and subsequent outflow obstruction, whereas the venous obstruction elevates hydrostatic pressure in the bowel wall because high pressure arterial inflow may continue despite venous occlusion. The vascular engorgement and oedema of the bowel wall in turn lead to leakage of extravascular fluid into the bowel wall and mesentery. The resultant oedematous bowel may have a “halo” or “target” appearance due to mild mucosal enhancement, submucosal and muscularis propria nonenhancement, and mild serosal/subserosal enhancement (Fig. 10) [41].

Contrarily, hyperenhancement of the bowel wall in shock bowel indicates neither hyperaemia nor hyperperfusion, but typically corresponds to prolonged enhancement of the bowel wall due to reduced arterial perfusion after vasospasm of the mesenteric arteries caused by the effects of angiotensin II or after reduced venous outflow due to contraction of mesenteric veins caused by the effects of adrenaline and noradrenaline (Fig. 9).

## Extra-bowel wall signs

### Fat stranding and ascites

Mesenteric fat stranding, mesenteric fluid and ascites are also nonspecific CT findings in acute bowel ischaemia, and their presence depends heavily on the cause, pathogenesis and severity of the ischaemia as well as on its location in the small or large bowel.

In exclusively arterial occlusive mesenteric ischaemia, the presence of segmental mesenteric fat stranding and free fluid interleaved between the mesenteric folds associated with the poorly enhancing bowel is highly suggestive of transmural infarction [42].

In venous outflow obstruction. The vascular engorgement and oedema of the bowel wall in turn lead to leakage of extravascular fluid into the bowel wall and mesentery (Fig. 7).

### Pneumatosis and portomesenteric gas

Pneumatosis and portomesenteric venous gas have been reported as less common but more specific findings of acute bowel ischaemia, being present in 6%–28 and 3%–14% of cases, respectively.

Although pneumatosis is not a specific finding of intestinal ischaemia, which may occur in a wide range of non-emergent benign scenarios, when found, bowel ischaemia must be excluded first and foremost.

**Fig. 11:**
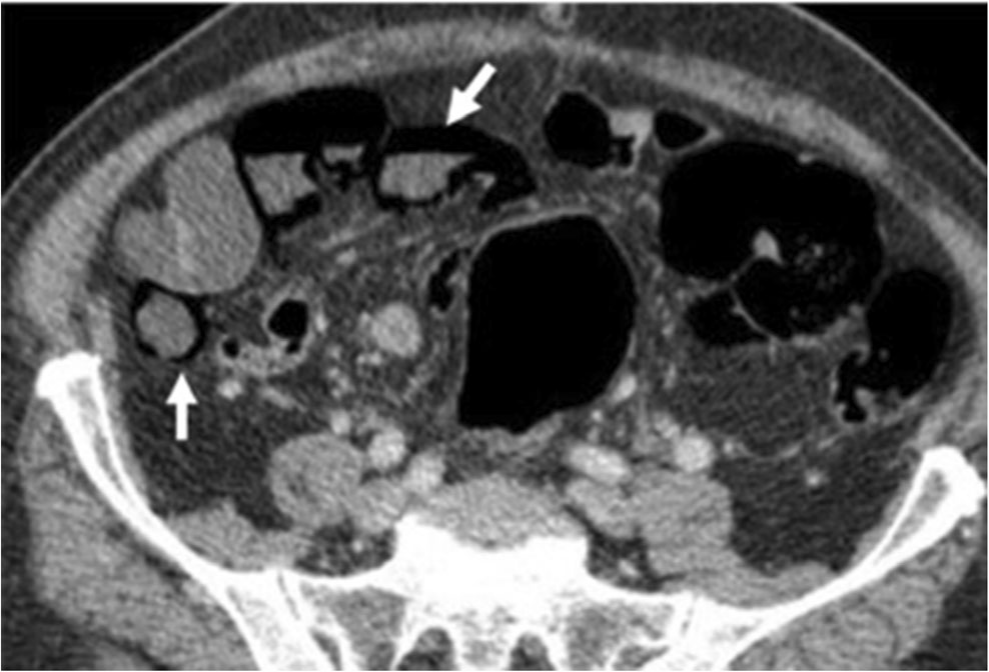
Pneumatosis. A 91-year-old male with mesenteric infarction. Contrast-enhanced CT images of the lower abdomen show gas in the bowel wall (white arrow)

Pneumatosis may manifest with small isolated gas bubbles in a circumferential distribution within an ischaemic bowel wall or as broad rims of air dissecting the entire bowel wall into two layers (Fig. 11) [42]. It should be differentiated from endoluminal gas, which usually presents in the least dependent position of the bowel lumen.

Portomesenteric venous gas may consist only of some small gaseous inclusions within the mesenteric veins or may extend into the intrahepatic branches of the portal vein, where it is typically found in the periphery of the liver (Fig. 5).

### Vessels

Blood clot or narrowing of the lumen of the visceral branches of the abdominal aorta or low-attenuation filling defect on contrast-enhanced CT of the superior mesenteric vein has higher specificity values (Fig. 2).

The calibre of the vessels is another important criterion. Due to the venous outflow obstruction, engorged mesenteric veins are typically observed (Fig. 10).

### Emboli involving other visceral organs

**Fig. 12:**
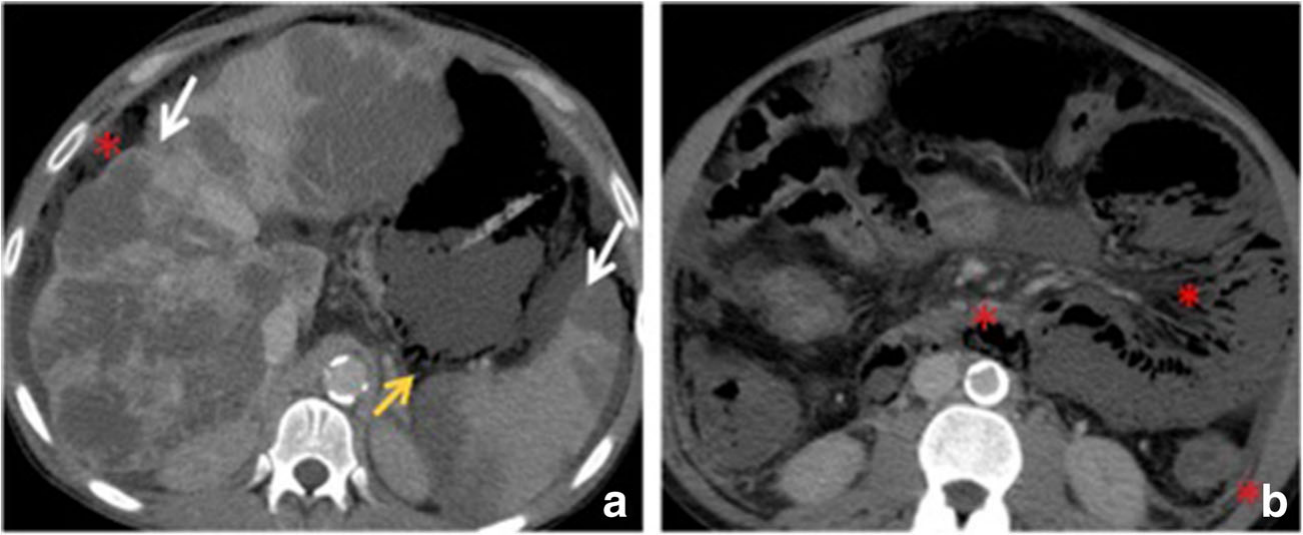
A 56-year-old male with mesenteric infarction. Axial enhanced CT shows various hypointense rounded regions of infarction in the hepatic parenchyma and wedge shaped in peripheral spleen parenchyma (white arrows) in a patient who has a mesenteric infarction. It also shows pneumatosis involving the gastric wall (orange arrow) and diffuse pneumo- and retropneumoperitoneum (*)

Infarcts of other visceral organs may suggest this diagnosis (Fig. 12).

### Pneumoperitoneum

While the severity of bowel ischaemia is variable, the presence of gas within the peritoneal cavity suggests perforation and peritonitis, which are high-mortality complications of infarction (Figs. 11 and 12).

## Prognosis

A number of scoring systems for predicting morbidity and mortality have been proposed for AMI, but these have not been validated in large-scale studies [41].

## Conclusion

Radiologists play a critical role in the diagnosis and appropriate triage of patients with mesenteric ischaemia. MDCT has a high specificity and sensitivity to diagnose intestinal ischaemia. Since mesenteric ischaemia may present with many different radiological appearances, an understanding of the intestinal vascular and mesenteric anatomy as well as the pathophysiology of mesenteric ischaemic disease helps improve the diagnosis of this challenging disease.
